# The role of firms in the economic assimilation of refugees

**DOI:** 10.23889/ijpds.v8i2.2335

**Published:** 2023-09-14

**Authors:** Hiromi Yumoto, Matt Cole, Ceren Ozgen, Liza Jabbour

**Affiliations:** 1 University of Birmingham, Birmingham, United Kingdom

## Objectives

To investigate the extent to which firm sorting effects influence the refugee-native earning gap in the Netherlands, and to examine the role of pay-setting effects in widening or narrowing the gap.

## Methods

Using Dutch wage and refugee registration data from 2014 to 2021, we employ a two-way effects model, also called the AKM model, and the Blinder-Oxaca decompose model, in order to decompose the refugee-native pay gap into firm sorting and pay-setting effects.

## Results

Our findings indicate that refugees in the Netherlands earn only a third of the average earnings of native workers and that 17% of the pay gap can be explained by firm sorting effects. Pay-setting effects are found to be negative, indicating that the pay-setting channel works to reduce pay gaps between natives and refugees. Limited access to higher-paying firms may lead to a mismatch between refugees' abilities and earnings, exacerbating the adverse impact of firm sorting.

## Conclusion

To promote refugee economic assimilation, higher-paying firms should provide accessible job opportunities based on fair assessments of refugees' skills. Our analysis underscores the importance of understanding and addressing the role of firm sorting in widening refugee-native pay gaps, and highlights the potential of pay-setting effects to reduce such gaps.

